# Establishment of an Assay with Ultrahigh Sensitivity for Detecting sEV-Derived PD-L1 as a Serum Biomarker for Lung Cancer—A Pilot Study Using TN-cyclon™

**DOI:** 10.3390/cimb47070564

**Published:** 2025-07-18

**Authors:** Kyo Okita, Hasumi Arita, Keita Sudo, Teruki Yoshimura, Etsuro Ito

**Affiliations:** 1 Department of Biology, TWIns, Waseda University, Shinjuku, Tokyo 162-0056, Japan; okita110513@akane.waseda.jp (K.O.); h4s1m9sango@ruri.waseda.jp (H.A.); kta-sudo2528@fuji.waseda.jp (K.S.); 2 R&D Department, BioPhenoMA Inc., Waseda University Entrepreneurship Center, Shinjuku, Tokyo 169-0051, Japan; yosimura@hoku-iryo-u.ac.jp; 3 School of Pharmaceutical Sciences, Health Sciences University of Hokkaido, Ishikari-Tobetsu, Hokkaido 061-0293, Japan; 4 Graduate Institute of Medicine, Kaohsiung Medical University, Sanmin, Kaohsiung 80708, Taiwan

**Keywords:** immune checkpoint, lung cancer, PD-L1, small extracellular vesicle, ultrasensitive ELISA

## Abstract

Programmed death-ligand 1 (PD-L1) is an immune checkpoint protein. The soluble form of PD-L1 (sPD-L1) and PD-L1 derived from small extracellular vesicles (sEVPD-L1) are promising cancer biomarkers. While sEVPD-L1 in particular may contribute to immune evasion and is associated with a poor prognosis, it exists only in trace amounts, making it difficult to detect using conventional enzyme-linked immunosorbent assay (ELISA) methods. Therefore, we developed an ultrasensitive detection method, TN-cyclon™. The TN-cyclon™ method combines sandwich ELISA with enzyme cycling amplification. We applied TN-cyclon™ to measure recombinant PD-L1 protein and sEVPD-L1 in serum samples from cancer patients and healthy donors. Recombinant PD-L1 protein was measured with an ultrasensitive detection limit of 0.172 pg/mL. In clinical specimens, sEVPD-L1 levels were significantly higher in lung cancer patients than in healthy donors, whereas sPD-L1 levels measured with a conventional ELISA did not differ significantly between groups. Our results demonstrated that the TN-cyclon™ method exhibits a 20-fold increase in sensitivity compared to a conventional ELISA. Although this is a pilot study, our new assay enables the detection of very low concentrations of sEVPD-L1 in serum that can be used to evaluate the predictive and prognostic performance of sEVPD-L1 in lung cancer patients in future studies.

## 1. Introduction

Cancer cells can express high levels of immune inhibitory signaling proteins. One of the most critical checkpoint pathways in this system is a tumor-induced immune suppression (immune checkpoint) mediated by the programmed cell death protein 1 (PD-1) and its ligand, programmed death ligand 1 (PD-L1) [1]. Cancer cells can exploit the PD-1/PD-L1 pathway to evade the immune system, contributing to cancer development and progression [2]. Therapies to inhibit this pathway, “immune checkpoint inhibitor therapy”, have been applied to various cancers in recent years, like non-small-cell lung cancer (NSCLC) and melanoma [2,3]. In previously treated patients with NSCLC (e.g., PD-L1-positive advanced NSCLC and advanced non-squamous NSCLC), however, the therapeutic effect of immune checkpoint inhibitors was limited, with the response rate remaining at ~20% [2,4]. Because treatment responses vary widely among patients, predictive biomarkers are essential for guiding treatment decisions.

Although the immunohistochemical (IHC) staining evaluation of PD-L1 is used as a companion diagnostic for immunotherapy, it is not an optimal biomarker [5]. Specifically, the cutoff value in IHC for establishing if immune checkpoint inhibitors can be used or not varies from test to test, and PD-L1 expression varies by site, even within the same tumor [5]. In this background, circulating PD-L1 has attracted increased attention in recent years. There are two types of circulating PD-L1: PD-L1 in small extracellular vesicles (sEVs) called exosomes (referred to as sEVPD-L1 in the present study) and soluble PD-L1 (sPD-L1) detected in the blood [6].

Previous studies have reached different conclusions regarding the usefulness of sPD-L1 as a prognostic biomarker. For example, some studies report that high sPD-L1 levels in patients with NSCLC and malignant melanoma are associated with a poorer prognosis, with similar results reported for gastric cancer [6,7]. On the other hand, some studies report that the sPD-L1 concentration is not significantly associated with patient prognosis. In early-stage NSCLC cases, plasma sPD-L1 levels did not correlate with recurrence risk or survival rate [8]. Further, changes in sPD-L1 levels during PD-1 inhibitor treatment do not significantly affect the prognosis of patients with advanced NSCLC [9]. Against this background of contradictory findings, sEVPD-L1 has gained more interest due to its association with poor prognosis in gastric cancer [10], highlighting the importance of measuring sEVPD-L1.

The detection of sEVPD-L1 using standard ELISA methods, however, is not easy as sEVs are present in extremely small quantities and the amount of PD-L1 protein they contain is also extremely small [11]. As Lu et al. pointed out, the detection limits of Western blotting and ELISA make them unsuitable for detecting low amounts of exoPD-L1 at an early cancer stage [11]. Although sEVPD-L1 measurement using digital ELISA (e.g., Simoa^TM^; Quanterix, Billerica, MA, USA) has been reported [12], this method requires specialized equipment and is therefore limited to research facilities and testing institutions. The development of a simple, easy-to-use, highly sensitive measurement method that can be used in standard laboratories and testing facilities is in high demand. To address this need, our group developed a new ultrasensitive immunoassay method called TN-cyclon^TM^ [13,14]. This method combines a sandwich ELISA method with an enzyme cycling method. In TN-cyclon^TM^, signal amplification proceeds in a triangular number pattern through repeated reactions of the enzyme 3α-hydroxysteroid dehydrogenase (HSD) with the coenzymes thio-nicotinamide adenine dinucleotide (NAD) and NADH. The results can be easily measured using a standard microplate reader.

In the present study, we aimed to measure sEVPD-L1 in lung cancer patients and healthy controls using the TN-cyclon^TM^ method, and to exhibit the usefulness of TN-cyclon^TM^ for assessing sEVPD-L1 as a biomarker. Ultrahigh sensitivity measurement means that the proteins in the collected solution can be measured even if the amount of collected solution is very small. In the present experiments, we applied TN-cyclon^TM^ to accurately quantify PD-L1 in an extremely small amount of collected sEVs. In addition, because we had a sufficient volume of sera from lung cancer patients and healthy controls, we measured sPD-L1 using a conventional ELISA method.

## 2. Materials and Methods

### 2.1. Patient Specimens

Sera from lung cancer patients were purchased from BizComJapan, Inc. (Tokyo, Japan) (for detailed information of samples, see Appendix A, Table A1). Sera from healthy controls were purchased from Central Link Co., Ltd. (Tokyo, Japan) (see Appendix A, Table A2). Specimens were obtained from patients with stage IVB lung cancer, the most severe cancer stage. All lung cancer patients and healthy controls were men in their 60s. According to the respective company datasheets, the sera from lung cancer patients were stored at −80 °C, and sera from healthy subjects were stored at −30 °C.

### 2.2. TN-cyclon^TM^ for Recombinant PD-L1

The TN-cyclon™ assay was originally developed in Ito’s laboratory [13,14]. The following reagents are currently commercially available from BioPhenoMA Inc. (PD-L1 TN-cyclon^TM^ ELISA kit; Tokyo, Japan). Briefly, each well of an Immuno Module F8 MaxiSorp plate (469949; Thermo Fisher Scientific, Waltham, MA, USA) was coated with 100 µL of capture antibody solution (mouse anti-human PD-L1 antibody, Clone A1-9F7-1, 1 µg/mL in 50 mM Na_2_CO_3_ buffer, pH 9.6; Hakarel Inc., Osaka, Japan) and the plates were incubated at 4 °C for over 16 h. Next, the wells were washed using a buffer containing 0.05% Tween-20 in Tris-buffered saline (TBS). To block nonspecific binding, 300 µL of 1% bovine serum albumin (BSA) in TBS was added to each well, followed by a 1 h incubation at room temperature. The plate was then washed with the same buffer before adding 100 µL of the PD-L1 antigen solution (human PD-L1 extracellular-domain protein; Hakarel Inc.). Antigens were prepared by two-fold serial dilution in 0.1% BSA/TBS to yield final concentrations ranging from 20 pg/mL to 0.625 pg/mL. Incubation was carried out at room temperature with shaking at approximately 400 rpm for 1 h. After the washes, a detection antibody (mouse anti-human PD-L1 antibody, Clone A1-13A11; Hakarel Inc.) conjugated with alkaline phosphatase (ALP) was used. The ALP conjugation was performed in-house using LK13 (Dojindo Laboratories, Kumamoto, Japan). The conjugated antibody was diluted to a final concentration of 200 ng/mL in TBS containing 0.1% BSA and 0.02% Tween-20, and 100 µL was added to each well. The plate was then incubated for 1 h at room temperature with shaking at approximately 400 rpm. After the washes, 100 µL of a thio-NAD cycling reaction mixture was added to each well to initiate signal amplification. The reaction mixture contained 100 mM tris-HCl (pH 9.0), 10 U/mL 3α-HSD, 0.4 mM 17β-methoxy-5β-androstan-3α-ol 3-phosphate, 1.0 mM NADH, and 2.0 mM thio-NAD. The accumulation of thio-NADH was monitored by measuring absorbance at 405 nm using a microplate reader (SH-1000; Corona Electric, Ibaraki, Japan). The absorbance at 405 nm was normalized to that at 660 nm, and the difference (A_405_ − A_660_) was used for statistical analysis.

### 2.3. Isolation of sEVs

sEVs were extracted from lung cancer patient and healthy control sera specimens using Total Exosome Isolation Reagent (from serum) (Thermo Fisher Scientific, Waltham, MA, USA). The specimens were centrifuged at 2000× *g* for 30 min at 4 °C to remove cells and cell debris. The reagent was added to the supernatant in an amount of 0.2 times the volume of the serum specimens and incubated at 4 °C for 30 min. The supernatant was then centrifuged at 10,000× *g* for 10 min at room temperature. The supernatant was discarded, and the pellet was suspended in 1 × TBS. The suspension was then filtered through a 0.22 µm filter to recover sEVs. The collected sEVs were stored at −80 °C.

### 2.4. Western Blotting

Western blotting was performed according to previous studies [15]. The sEV protein concentration was measured using a bicinchoninic acid protein assay (BCA) kit (23227; Thermo Fisher Scientific). The total protein amount (20 µg) was separated on a 12.5% acrylamide gel and transferred to a polyvinylidene difluoride membrane. The membrane was blocked for 1 h at room temperature with 3% BSA in Tween-20-added TBS. The primary antibody (CD81 antibody, Cat. No. 66866-1-Ig; Proteintech, Rosemont, IL, USA) was used at 1:1000 dilution in 3% BSA in Tween-20-added TBS and kept overnight at 4 °C. Horseradish peroxidase (HRP)-labeled secondary antibody [HRP-conjugated goat anti-mouse IgG(H+L), Cat. No. SA00001-1; Proteintech] was incubated for 1 h at room temperature. Novex™ ECL Chemiluminescent Substrate (Thermo Fisher Scientific) was used as the substrate to detect the bands. Density measurements were performed using LAS-3000 (Fujifilm, Tokyo, Japan). Images were analyzed using ImageJ (Ver. 1.54, NIH, Bethesda, MD, USA).

### 2.5. Measurement of sEV Size

The sEV suspension was prepared in 1 × TBS to a concentration of 34 μg/100 µL or more per total protein amount. The dynamic light scattering (DLS) method was used to examine the size of collected sEVs. DLS measurements were performed with a ZetaSizer nano S90 (Malvern Panalytical, Malvern, UK). Then, 50 µL was transferred to a disposable cuvette for size measurements. The measurements consisted of 20 repeated 10 s runs. The data were processed by ZetaSizer nano S90 analytical software (Ver. 8.02).

### 2.6. Measurement of sEVPD-L1

sEVPD-L1 levels were measured using the PD-L1 TN-cyclon^TM^ ELISA kit (BioPhenoMA Inc.). This method allows for the measurement of extremely small amounts of PD-L1 protein. The same antibodies used to measure the recombinant PD-L1 protein (see Section 2.2) were also used to measure sEVPD-L1. The total sEV protein concentration extracted from the specimens was adjusted to 125 µg/mL using a BCA kit (Thermo Fisher Scientific), and Triton X-100 was added to a final concentration of 1%.

### 2.7. Measurement of sPD-L1

sPD-L1 was measured using a commercially available ELISA kit (PD-L1 ELISA kit, Human, HAK-HELPDL1-1; Hakarel Inc.). The same antibodies used to measure the recombinant PD-L1 protein (see Section 2.2) were also used to measure sPD-L1. Therefore, it is possible to compare the results of this sPD-L1 measurement with the sEVPD-L1 measurement described above (see Section 2.6). According to the ELISA kit instructions, the serum specimens were measured without dilution.

### 2.8. Statistical Analysis

For more than two groups, one-way ANOVA and pairwise *t*-tests corrected by the Holm method were used to evaluate significant differences using R (version 4.2.2; The R Foundation for Statistical Computing, Vienna, Austria). A Wilcoxon rank-sum test was used to compare between two groups. *p* values of 0.05 or less were considered statistically significant. The limit of detection (LOD) and the limit of quantification (LOQ) of the TN-cyclon™ method were estimated using a confidence factor of 3 and 10, respectively, of the blank standard deviation. The coefficient of variation (CV) was calculated as the standard deviation divided by the mean and expressed as a percentage.

## 3. Results

### 3.1. Recombinant PD-L1 Measurements Using TN-cyclon^TM^

**Figure 1:**
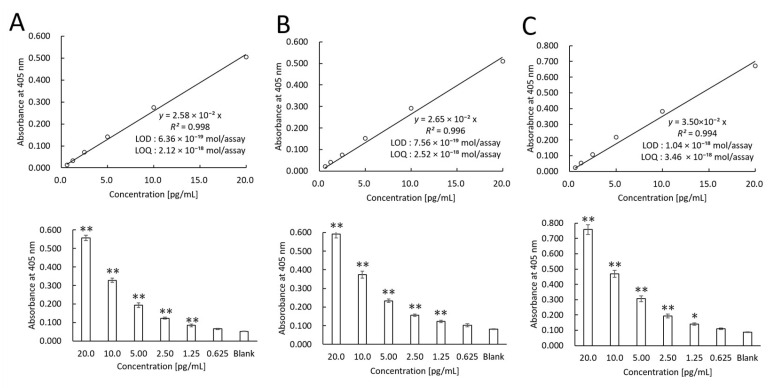
Linear calibration curves of TN-cyclon™ for PD-L1 recombinant protein. The experiments were conducted by three different experimenters as indicated by (**A**–**C**). Absorbance was measured at the following time points of thio-NAD cycling: 45 min for Experimenter A, 60 min for Experimenter B, and 60 min for Experimenter C. The measurement time was selected from each record to provide the most sensitive measurement. The lower panels show that data are presented as the mean ± SD. * *p* < 0.05 and ** *p* < 0.01.

The LOD of recombinant PD-L1 protein was examined. We also examined reproducibility by different experimenters. The linear calibration curves created by three different experimenters were as follows (Figure 1): Experimenter 1—*y* = 2.58 × 10^−2^*x* (*R*^2^ = 0.998) (Figure 1A); Experimenter 2—*y* = 2.65 × 10^−2^*x* (*R*^2^ = 0.996) (Figure 1B); and Experimenter 3—*y* = 3.50 × 10^−2^*x* (*R*^2^ = 0.994) (Figure 1C).

The LODs were calculated from the slope of the linear calibration curves using a standard deviation (SD) and a confidence factor of 3: 0.172 pg/mL (Figure 1A), 0.204 pg/mL (Figure 1B), and 0.280 pg/mL (Figure 1C). Considering that the well volume of this assay was 100 µL/well and the molecular weight of the PD-L1 recombinant protein was 27 kDa, the LODs were 6.36 × 10^−19^ moles/assay, 7.56 × 10^−19^ moles/assay, and 1.04 × 10^−18^ moles/assay, respectively. The LOQs were calculated from the slope of the linear calibration curve using the SD and a confidence factor of 10: 0.572 pg/mL (2.12 × 10^−18^ moles/assay) (Figure 1A), 0.680 pg/mL (2.52 × 10^−18^ moles/assay) (Figure 1B), and 0.934 pg/mL (3.46 × 10^−18^ mol/assay) (Figure 1C).

The intra-assay CVs were examined. Figure 1 shows that the difference from the blank value occurred at a recombinant PD-L1 concentration of 1.25 pg/mL. Thus, the intra-assay CVs were 8.54% (Figure 1A), 4.97% (Figure 1B), and 5.31% (Figure 1C). In addition, the inter-assay CVs were examined. The inter-assay CV at the recombinant PD-L1 concentration of 1.25 pg/mL was 5.50% when the thio-NAD cycling was performed at 60 min.

### 3.2. Characteristics of sEVs

**Figure 2:**
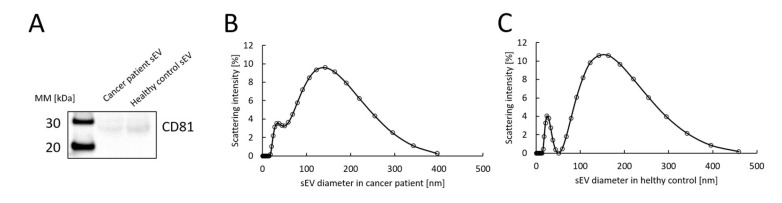
Characteristics of sEVs collected from the sera obtained from lung cancer patients and healthy subjects. (**A**) Western blotting of an sEV marker, CD81. (**B**) Particle size measurement of sEV collected from the serum of a lung cancer patient. (**C**) Particle size measurement of sEV collected from the serum of a healthy subject.

sEVs were collected from sera obtained from lung cancer patients and healthy subjects using polymer precipitation and a 0.22 µm filter. The expression of an EV marker, CD81, was confirmed by Western blotting (Figure 2A). CD81 is a known marker of sEVs [16], and its molecular weight is approximately 20–30 kDa. In the present study, we observed a band around 20–30 kDa corresponding to CD81.

The sEV particle size in the sera from a lung cancer patient (Figure 2B) and from a healthy control (Figure 2C) was examined using a Zetasizer. The mean diameter of particles detected in a lung cancer patient was 85.65 nm and that in a healthy control was 84.73 nm. Based on the size, we judged the particles collected by us to be sEVs.

### 3.3. Measurements of sEVPD-L1 and sPD-L1 Derived from Sera Obtained from Lung Cancer Patients and Healthy Controls

**Figure 3:**
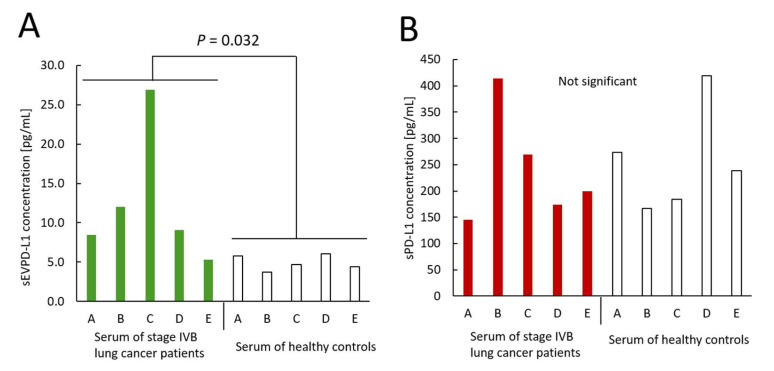
Concentrations of sEVPD-L1 and sPD-L1. Sera from five lung cancer patients and sera from five healthy controls were examined. (**A**) Concentration of sEVPD-L1. Green bars show the data of lung cancer patients. (**B**) Concentration of sPD-L1. Red bars show the data of lung cancer patients. The sEVPD-L1 concentration was higher in lung cancer patients (stage IVB) than in healthy controls.

We used five lung cancer patient specimens and five healthy subject specimens. sEVPD-L1 levels in sera collected from lung cancer patients were 8.48 pg/mL, 12.0 pg/mL, 26.9 pg/mL, 9.07 pg/mL, and 5.28 pg/mL, respectively (Figure 3A). On the other hand, sEVPD-L1 levels in sera collected from the healthy control group were 5.82 pg/mL, 3.76 pg/mL, 4.72 pg/mL, 6.07 pg/mL, and 4.42 pg/mL, respectively (Figure 3A). The difference in the sEVPD-L1 levels between the lung cancer patient group and the healthy control group was statistically significant (*p* = 0.032).

sPD-L1 levels in the lung cancer patient specimens were 146 pg/mL, 414 pg/mL, 269 pg/mL, 174 pg/mL, and 199 pg/mL, respectively, whereas the sPD-L1 results for the healthy control specimens were 274 pg/mL, 167 pg/mL, 184 pg/mL, 419 pg/mL, and 239 pg/mL, respectively. No statistically significant difference in the sPD-L1 amounts was detected between the lung cancer patients and healthy controls.

## 4. Discussion

We successfully developed an ultrasensitive method for measuring PD-L1 using the TN-cyclon^TM^ method. Our results using this method revealed that our new assay enables the detection of very low concentrations of sEVPD-L1 in serum that can be used to evaluate the predictive and prognostic performance of sEVPD-L1 in lung cancer patients in future studies. The LOD of recombinant PD-L1 protein was 0.172 pg/mL. This sensitivity approaches the values (LOD = 0.044–0.05 pg/mL) reported using the Simoa^TM^ technology (Quanterix) [17] and is much greater than that of conventional ELISA (LOD > 3 pg/mL) [18,19].

Most previous studies have used standard ELISA methods for detecting sEVPD-L1, but the results are unreliable because of their poor measurement sensitivity [11,18,19]. Although a few studies have utilized Simoa^TM^ [12], which provides reliable results, the Simoa^TM^ technique requires expensive equipment and is difficult to apply for clinical research. In contrast, the TN-cyclon™ method can be used with a standard microplate reader, simplifying measurements of low levels of PD-L1 [13,14,15].

In the present study, we also measured sEVPD-L1 and sPD-L1 in sera obtained from lung cancer patients and healthy controls. The sEVPD-L1 concentrations in sera obtained from lung cancer patients and healthy controls were 42.2–215.2 pg/mg and 30.08–48.56 pg/mg, respectively, when calculated based on 1 mg of total protein. These values are higher than previously reported values. For example, Li et al., using a commercially available ELISA kit, reported 5.17 pg/mg in lung cancer patients (stage III, IV) and 1.84 pg/mg in healthy controls [20]. The ELISA kit used by Li et al. differs substantially from our in-house ultrasensitive ELISA technology [20]. Although differences in measurement techniques may explain the observed variability, increasing a larger sample size is essential for drawing more reliable conclusions.

On the other hand, we found no significant difference in serum sPD-L1 concentrations between lung cancer patients and healthy controls in the present study. Some previous studies also reported no significant difference in sPD-L1 concentrations between lung cancer patients and healthy controls [7,21]. Other studies reported that the average sPD-L1 concentration in the sera of lung cancer patients (stage IV) was 248 pg/mL [22], which is in good agreement with the present findings (mean 240 pg/mL), whereas the average sPD-L1 concentration in the sera of healthy controls was 50–150 pg/mL [21], a lower value than our result (mean 256.6 pg/mL).

Whether sEVPD-L1 or sPD-L1 is a more useful biomarker is under debate. sEVPD-L1 is expected to be a more useful biomarker because it is secreted in large amounts into the circulation, where it induces systemic immunosuppression, and because sEVs have a small volume and large specific surface area, allowing them to penetrate deep into tissues [23]. Some clinical studies demonstrated that PD-L1 expression on tumor cells or in the tumor microenvironment positively correlates with the response rate of anti-PD-1/PD-L1 therapy, indicating that PD-L1 may serve as a potential selection marker for patient stratification [5]. On the other hand, Li et al. [20] demonstrated that the sEVPD-L1 levels were significantly higher in patients with stage III and IV NSCLC than in healthy controls, whereas there was no difference in the levels of sPD-L1. Our present results are consistent with the findings reported by Li et al.

Other reports, however, suggest that sPD-L1 is a better biomarker than sEVPD-L1. Oh et al. reported that high sPD-L1 levels are associated with poor prognosis, and that sPD-L1 can be used as a biomarker for prognostic evaluation [7]. In addition, Ancin et al. reported that the concentration of sPD-L1 was significantly higher in NSCLC patients compared to the control group [24]. Other investigators argue that the balance of PD-1 expression on tumor-infiltrating effector T cells and regulatory T cells correlates with the therapeutic effect of PD-1/PD-L1 inhibitor therapy and may predict the therapeutic effect with high accuracy [25]. This biomarker was also validated in specimens from patients with advanced solid malignant tumors other than lung and gastric cancers who were treated with PD-1/PD-L1 inhibitor. The analysis confirmed that PD-1/PD-L1 inhibitor therapy was more effective in cases with a higher PD-1 expression on effector T cells and a lower PD-1 expression on regulatory T cells.

## 5. Conclusions

We demonstrated that an immune checkpoint molecule, PD-L1, can be measured with ultrahigh sensitivity by simply applying the colorimetric method TN-cyclon^TM^ without the use of complicated equipment. The TN-cyclon^TM^ method allowed us to measure the PD-L1 concentration in sEVs contained in sera obtained from lung cancer patients (stage IVB). As this is only a pilot study, we plan to validate our findings in a larger patient cohort and the patients with different stages to provide more definitive evidence. Further discussion is needed to identify the most informative protein markers and to determine whether liquid biopsy is an effective method for assessing circulating PD-1/PD-L1 as a predictive biomarker of the efficacy of immune checkpoint inhibitors.

## Data Availability

The data supporting the findings of this study are available within the publication and upon reasonable request to the corresponding author.

## Abbreviations

The following abbreviations are used in this manuscript:

BCA	Bicinchoninic acid
CV	Coefficient of variation
DLS	Dynamic light scattering
ELISA	Enzyme-linked immunosorbent assay
HSD	Hydroxysteroid dehydrogenase
IHC	Immunohistochemistry
LOD	Limit of detection
LOQ	Limit of quantification
NSCLC	Non-small-cell lung cancer
PD-1	Programmed death-1
PD-L1	Programmed death ligand-1
sEV	Small extracellular vesicle
sPD-L1	Soluble form of PD-L1
sEVPD-L1	PD-L1 in small extracellular vesicles
TN-cyclon^TM^	Ultrasensitive ELISA combined with thio-NAD cycling

## Appendix A

**Table A1:** Information of lung cancer patients.

Sample ID	Donation Date	Gender	Age	Ethnicity	Location	Histological Diagnosis	TNM	Stage	Smoking History	Family History	Medical History	Treatment	Date of Surgery
A	13 March 2023	M	62	Caucasian	lower lobe of the left lung, brain and bones mets	adenocarcinoma	T4N2M1c	IVB	no	grandmother- gastric cancer	hypertension	no	no
B	10 May 2023	M	66	Caucasian	upper lobe of the right lung, bones, brain mets	adenocarcinoma	T4N2M1c	IVB	45 years 1 pack/day	no	varicose veins	no	no
C	11 August 2023	M	60	Caucasian	upper lobe of the left lung, adrenal gland and brain mets	adenocarcinoma	T4N2M1c	IVB	no	no	hypertension, diabetes mellitus type 2, chronic bronchitis	no	no
D	13 September 2023	M	60	Caucasian	upper lobe of the right lung, brain and bone mets	adenocarcinoma	T4NxM1c	IVB	40 years 1pack/day	no	chronic gastritis, thymus cyst, chronic bronchitis	no	13 Sep-tember 2023
E	5 December 2023	M	68	Caucasian	lower lobe of the left lung, right lung, brain mets	adenocarcinoma	T4N3M1c	IVB	40 years 1 pack/day	no	chronic gastritis	4 December 2023—1 course paclitaxel, carboplatin	no

**Table A2:** Information of healthy controls.

Sample ID	Donation Date	Blood Type	Gender	Age	Ethnicity
A	31 July 2023	A+	M	68	Caucasian
B	8 August 2023	A+	M	66	Caucasian
C	31 July 2023	A+	M	62	Caucasian
D	6 August 2023	A+	M	60	Caucasian
E	31 July 2023	A+	M	60	Caucasian

## References

[1] Dermani F.K., Samadi P., Rahmani G., Kohlan A.K., Najafi R. (2019). PD-1/PD-L1 immune checkpoint: Potential target for cancer therapy. J. Cell. Physiol..

[2] Borghaei H., Paz-Ares L., Horn L., Spigel D.R., Steins M., Ready N.E., Chow L.Q., Vokes E.E., Felip E., Holgado E. (2015). Nivolumab versus docetaxel in advanced nonsquamous non-small-cell lung cancer. N. Engl. J. Med..

[3] Topalian S.L., Hodi F.S., Brahmer J.R., Gettinger S.N., Smith D.C., McDermott D.F., Powderly J.D., Carvajal R.D., Sosman J.A., Atkins M.B. (2012). Safety, activity, and immune correlates of anti-PD-1 antibody in cancer. N. Engl. J. Med..

[4] Herbst R.S., Baas P., Kim D.W., Felip E., Pérez-Gracia J.L., Han J.Y., Molina J., Kim J.H., Arvis C.D., Ahn M.J. (2016). Pembrolizumab versus docetaxel for previously treated, PD-L1-positive, advanced non-small-cell lung cancer (KEYNOTE-010): A randomised controlled trial. Lancet.

[5] Patel S.P., Kurzrock R. (2015). PD-L1 expression as a predictive biomarker in cancer immunotherapy. Mol. Cancer Ther..

[6] Széles Á., Fazekas T., Váncsa S., Váradi M., Kovács P.T., Krafft U., Grünwald V., Hadaschik B., Csizmarik A., Hegyi P. (2023). Pre-treatment soluble PD-L1 as a predictor of overall survival for immune checkpoint inhibitor therapy: A systematic review and meta-analysis. Cancer Immunol. Immunother..

[7] Oh S.Y., Kim S., Keam B., Kim T.M., Kim D.W., Heo D.S. (2021). Soluble PD-L1 is a predictive and prognostic biomarker in advanced cancer patients who receive immune checkpoint blockade treatment. Sci. Rep..

[8] Mildner F.O., Sykora M.M., Hackl H., Amann A., Zelger B., Sprung S., Buch M.L., Nocera F., Moser P., Maier H. (2024). Soluble PD-L1 shows no association to relapse and overall survival in early stage non-small cell lung cancer (NSCLC). Lung Cancer.

[9] Shimizu T., Inoue E., Ohkuma R., Kobayashi S., Tsunoda T., Wada S. (2023). Soluble PD-L1 changes in advanced non-small cell lung cancer patients treated with PD-1 inhibitors: An individual patient data meta-analysis. Front. Immunol..

[10] Fan Y., Che X., Qu J., Hou K., Wen T., Li Z., Li C., Wang S., Xu L., Liu Y. (2019). Exosomal PD-L1 retains immunosuppressive activity and is associated with gastric cancer prognosis. Ann. Surg. Oncol..

[11] Lu M.M., Yang Y. (2024). Exosomal PD-L1 in cancer and other fields: Recent advances and perspectives. Front. Immunol..

[12] Li J.W., Wei P., Guo Y., Shi D., Yu B.H., Su Y.F., Li X.Q., Zhou X.Y. (2020). Clinical significance of circulating exosomal PD-L1 and soluble PD-L1 in extranodal NK/T-cell lymphoma, nasal-type. Am. J. Cancer Res..

[13] Kobayashi Y., Kyosei Y., Ogawa R., Okita K., Yoshimura T., Ito E. (2024). Ultrasensitive protein-level detection for respiratory infectious viruses. Front. Immunol..

[14] Makioka D., Inada M., Awano M., Saito E., Shinoda T., Abe S., Yoshimura T., Müller M., Sasagawa T., Ito E. (2024). Quantification of HPV16 E7 oncoproteins in urine specimens from women with cervical intraepithelial neoplasia. Microorganisms.

[15] Tsurusawa N., Iha K., Sato A., Tsai H.Y., Sonoda H., Watabe S., Yoshimura T., Wu D.C., Lin M.W., Ito E. (2022). Ultrasensitive detection of GRP78 in exosomes and observation of migration and proliferation of cancer cells by application of GRP78-containing exosomes. Cancers.

[16] Signorelli D., Ghidotti P., Proto C., Brambilla M., De Toma A., Ferrara R., Galli G., Ganzinelli M., Lo Russo G., Prelaj A. (2022). Circulating CD81-expressing extracellular vesicles as biomarkers of response for immune-checkpoint inhibitors in advanced NSCLC. Front. Immunol..

[17] Quanterix Corporation Simoa PD-L1 Data Sheet HD-1/HD-X Rev03. https://www.quanterix.com/wp-content/uploads/2020/12/Simoa_PD-L1_Data_Sheet_HD-1_HD-X_Rev03.pdf.

[18] Abcam Human PD-L1 ELISA Kit [28-8]. https://www.abcam.com/en-us/products/elisa-kits/human-pd-l1-elisa-kit-28-8-ab277712.

[19] Proteintech Human PD-L1 ELISA Kit (KE00074). https://www.ptglab.com/products/Human-PD-L1-ELISA-Kit-KE00074.htm.

[20] Li C., Li C., Zhi C., Liang W., Wang X., Chen X., Lv T., Shen Q., Song Y., Lin D. (2019). Clinical significance of PD-L1 expression in serum-derived exosomes in NSCLC patients. J. Transl. Med..

[21] Ćeriman Krstić V., Jovanović D., Samardžić N., Gajić M., Kotur Stevuljević J., Klisic A., Soldatović I., Radončić D., Roksandić Milenković M., Šeha B. (2025). The potential role of sPD-L1 as a predictive biomarker in EGFR-positive non-small-cell lung cancer. Curr. Issues Mol. Biol..

[22] Sasaki T., Nonomura R., Tabata T., Yoshimura N., Hata S., Shimada H., Nakamura Y. (2023). Study of the clinicopathological features of soluble PD-L1 in lung cancer patients. J. Rural Med..

[23] Yu Z.L., Liu J.Y., Chen G. (2022). Small extracellular vesicle PD-L1 in cancer: The knowns and unknowns. NPJ Precis. Oncol..

[24] Ancın B., Özercan M.M., Yılmaz Y.M., Uysal S., Kumbasar U., Sarıbaş Z., Dikmen E., Doğan R., Demircin M. (2022). The correlation of serum sPD-1 and sPD-L1 levels with clinical, pathological characteristics and lymph node metastasis in nonsmall cell lung cancer patients. Turk. J. Med. Sci..

[25] Kumagai S., Togashi Y., Kamada T., Sugiyama E., Nishinakamura H., Takeuchi Y., Vitaly K., Itahashi K., Maeda Y., Matsui S. (2020). The PD-1 expression balance between effector and regulatory T cells predicts the clinical efficacy of PD-1 blockade therapies. Nat. Immunol..

